# Integrating Traditional Medicine into Modern Inflammatory Diseases Care: Multitargeting by *Rhus verniciflua* Stokes

**DOI:** 10.1155/2014/154561

**Published:** 2014-06-12

**Authors:** Ji Hye Kim, Yong Cheol Shin, Seong-Gyu Ko

**Affiliations:** Laboratory of Clinical Biology and Pharmacogenomics, Department of Preventive Medicine, College of Oriental Medicine, Kyunghee University, 1 Hoegi-dong, Seoul 130-701, Republic of Korea

## Abstract

Despite the fact that numerous researches were performed on prevention and treatment of inflammation related diseases, the overall incidence has not changed remarkably. This requires new approaches to overcome inflammation mediated diseases, and thus traditional medicine could be an efficacious source for prevention and treatment of these diseases. In this review, we discuss the contribution of traditional medicine, especially *Rhus verniciflua* Stokes, to modern medicine against diverse inflammation mediated diseases. Traditionally, this remedy has been used in Eastern Asia for the treatment of gastric problems, hepatic disorders, infectious diseases, and blood disorders. Modern science has provided the scientific basis for the use of *Rhus verniciflua* Stokes against such disorders and diseases. Various chemical constituents have been identified from this plant, including phenolic acid, and flavonoids. Cell-based studies have exhibited the potential of this as antibacterial, antioxidant, neuroprotective, anti-inflammatory, growth inhibitory, and anticancer activities. Enormous animal studies have shown the potential of this against proinflammatory diseases, neurodegenerative diseases, diabetes, liver diseases, and chemical insults. At the molecular level, this medicinal plant has been shown to modulate diverse cell-signaling pathways. In clinical studies, *Rhus verniciflua* Stokes has shown efficacy against various cancer patients such as colorectal, gastric, hepatic, renal, pancreatic, and pulmonary cancers. Thus, this remedy is now exhibiting activities in the clinic.

## 1. Introduction

Inflammation is an essential part of the body's natural responses against harmful stimuli, such as pathogens, toxin, damaged cells, irritants, stress, or injury. Initially, although the symptoms of acute inflammation are unpleasant, they are absolutely necessary for the healing processes. However, sometimes inflammation can cause further inflammation (chronic inflammation), which can last for several months and even years. It can result from failure to eliminate an acute inflammation, an autoimmune response to a self-antigen. Chronic inflammation can eventually cause several diseases and conditions, including some cancers, asthma, rheumatoid arthritis, atherosclerosis, periodontitis, ischemic heart disease, and ulcerative colitis. Therefore, inflammation needs to be well regulated [1].

**Figure 1:**
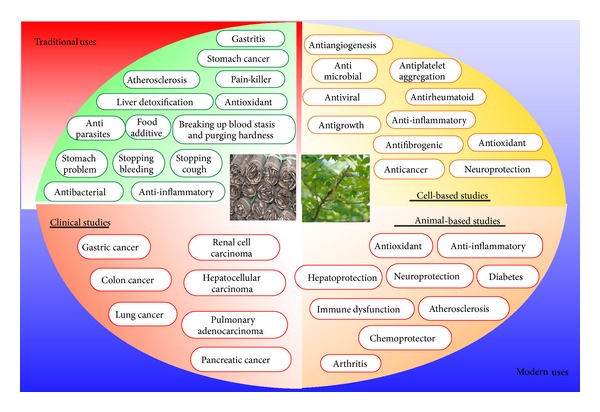
Schematic representation for the traditional and modern uses of* Rhus verniciflua* Stokes.

Traditional medicine is a part of traditional East Asian medical systems and has been used for treating various kinds of diseases including cancer for thousands of years, and, recently, increasing emphasis has been focused on the research on traditional medicine. Particularly, many herbs and medicinal plants have been reported to prevent and inhibit various kinds of diseases [2, 3]. Many traditional medicines and their natural products in eastern countries are relatively low priced, are efficacious resources for new drug discovery, and show very little adverse effects identified in clinical research. One of the remedies is* Toxicodendron vernicifluum, *formerly* Rhus verniciflua* Stokes, which has been used for thousands of years, mostly in Asian countries.* Rhus verniciflua* Stokes is an Asian tree species of genus* Toxicodendron*, which belongs to Anacardiaceae family, and is cultivated in regions of China, Korea, and Japan [4].* Rhus verniciflua* Stokes has a long tradition of use in Eastern Asian medical systems. This remedy has been used for enormous purposes since ancient times. In Korea,* Rhus verniciflua* Stokes has been used as an herbal therapy for the treatment of abdominal masses since the 15th century AD [5]. This was used to relieve stomach problems and liver detoxification and to stop bleeding and cough. It also has been used for digestive problems such as gastritis, helping to break up blood stasis, and purging hardness. It also helps to relieve pain.* Rhus verniciflua* Stokes has been used as a food additive as well. However, scientific evidence proving these health benefits of* Rhus verniciflua* Stokes is lacking.* In vitro* studies of this remedy have shown potential of antibacterial, antimicrobial, antirheumatoid, anti-inflammatory, antioxidant, antigrowth, neuroprotective, antiplatelet aggregation, and anticancer activities (Figure 1). In* in vivo* studies, this remedy exhibits activities against inflammatory conditions, neurodegenerative diseases, liver problem, diabetes, arthritis, and atherosclerosis. It has also been shown to protect from numerous chemical insults. Some clinical researches have already evaluated the safety and efficacy of* Rhus verniciflua* Stokes against cancer patients. In the following sections, we provide the evidence for the biological activities of* Rhus verniciflua* Stokes from preclinical studies. The common chemical entities isolated from* Rhus verniciflua* Stokes are also discussed.

## 2. Preclinical Studies with* Rhus verniciflua* Stokes

Numerous researches from both* in vitro* and* in vivo* studies have indicated the activities of* Rhus verniciflua* Stokes against numerous diseases. In this section, we provide evidence from* in vitro* and* in vivo* studies for the biological activities of* Rhus verniciflua* Stokes (Tables 1 and 3).

### 2.1. Cell-Based Studies

#### 2.1.1. Antibacterial Activity

*Rhus verniciflua* Stokes possesses antibacterial activity. In one study, antibacterial activity of the urushiol, major component of the remedy against* Helicobacter pylori* (*H. pylori*), was investigated. All 3 strains,* H. pylori* NCTC 11637,* H. pylori* 69, and* H. pylori* 219, survived within pH 6.0–9.0. The minimal inhibitory concentrations (MIC) of the extract against strains ranged 0.064–0.256 mg/mL [6].

#### 2.1.2. Anticancer Activity

Carcinogenesis is a multistep process involving the transformation, survival, proliferation, angiogenesis, invasion, and metastasis of the tumor and may take over 30 years [40]. Modern science has defined that cancer is a hyperproliferative disorder that involves sustaining proliferative signaling, evading growth suppressors, resisting cell death, inducing angiogenesis, activating invasion and metastasis, and enabling replicative immortality [41]. Extensive research has also demonstrated the biology of cancer. Biological cancer target therapy is those cell signaling pathways, including survival signaling (e.g., phosphatidylinositide 3-kinases- (PI3K-) Akt/protein kinase B (PKB)); cell cycle proteins (e.g., p53, cyclins, and cell cycle dependent kinase inhibitors (CDKIs)); angiogenesis (e.g., vascular endothelial growth factor (VEGF)); and antiapoptosis (e.g., B-cell lymphoma 2 (bcl-2), B-cell lymphoma-extra large (bcl-X_L_), X-linked inhibitor of apoptosis protein (XIAP), survivin, and FLICE-like inhibitory protein (FLIP)) [2].

*Rhus verniciflua* Stokes has been most widely investigated for its anticancer activity. The most common cancer types in which* Rhus verniciflua* Stokes has shown potential are those of the liver, blood, breast, bone, and stomach (Table 1). In stomach carcinoma cell model, an ethanol extract of* Rhus verniciflua* Stokes significantly inhibited G_1_ cell cycle progression* via *p27^Kip1^ CDKI upregulation and induced mitochondrial apoptosis through the increment of Bax expression, the inhibition of Bcl-2 expression, the release of cytochrome* c,* and the activation of caspase-3 and caspase-9 cascade, and this mechanism by* Rhus verniciflua* Stokes was an enhanced inhibition of the PI3K-Akt/PKB survival pathway [11, 13]. One study concluded that an ethanol extract of* Rhus verniciflua* Stokes has the potential to induce apoptosis, based on the increase in DNA fragmentation in human B and T lymphoma cell lines, BJAB, and Jurkat [8]. The anticancer activity of* Rhus verniciflua* Stokes has been shown in human osteosarcoma cells as well. One study investigated the apoptotic effects of* Rhus verniciflua* Stokes chloroform-methanol fraction from an acetone extract (RCMF) on human osteosarcoma (HOS) cells [9]. PARP cleavage was closely associated with the RCMF-induced apoptosis in HOS cells. Furthermore, the activation of caspase-8 and Bax, the inhibition of Bcl-2 expression, and the release of cytochrome *c* are shown to be involved in the RCMF-mediated apoptosis. Some other studies using cell line model have also shown the potential of* Rhus verniciflua* Stokes against various kinds of cancers [14]. Interestingly, among the 4 fractions: diethyl ether, ethyl-acetate (EtOAC), butanol, and water fraction, the EtOAC fraction from* Rhus verniciflua* Stokes extract contained highly concentrated phenolic compounds, had the most cytotoxic effect in gastric, breast, liver, lung and colon cancer, particularly effective against gastric and breast cancer cells. As well as the EtOAC fraction showed a stronger apoptotic effect on these cells.

#### 2.1.3. Anti-Inflammatory Activity

Chronic inflammation has been associated with numerous human chronic diseases, including cardiovascular, pulmonary, autoimmune, and degenerative diseases, cancer, and diabetes [42]. Many researchers have reported that* Rhus verniciflua* Stokes possesses an anti-inflammatory effect by modulating the expression of proinflammatory mediators. For example,* Rhus verniciflua* Stokes exhibited anti-inflammatory activity during endotoxin, lipopolysaccharide (LPS) infection in Raw264.7 macrophage [17–19, 22]. Raw264.7 macrophage is a well-characterized inflammatory model induced by LPS.* Rhus verniciflua* Stokes showed its activity by suppression of inducible nitric oxide synthase (iNOS) and cyclooxygenase-2 (COX-2) expression, which results in inhibiting nitric oxide (NO) and prostaglandin E_2_ (PGE_2_) production. All researches studied different kinds of extraction or fractionation methods; for instance, the Jung et al. group used 80% ethanol* Rhus verniciflua* Stokes extract without nonionic compounds, the Oh et al. group used water extract, another Jung group used n-butanol fraction from 80% ethanol* Rhus verniciflua* Stokes extract, and the other group used crude 80% ethanol* Rhus verniciflua* Stokes extract, but the results showed the same effect. Early allergic inflammation is one of the more prominent inflammatory responses and is characterized by the release of histamine and mast cell granule proteins by degranulation, as well as the production of leukotrienes, prostaglandins, and cytokines [43]. One study evaluated the effects of* Rhus verniciflua* Stokes against phorbol myristate acetate (PMA) and calcium ionophore A23187-induced mast cell activation [20]. The treatment of* Rhus verniciflua* Stokes significantly modulated the expressions of signal molecules related to allergic inflammatory responses* via* the extracellular signal-regulated kinases (ERK) signaling pathway and inhibited the expressions of inflammation related cytokines—[tumor necrosis factor (TNF)-*α*, interleukin (IL)-6, and IL-8] that were stimulated by the treatment with both PMA and A23187.* Rhus verniciflua* Stokes inhibited the nuclear translocation of nuclear factor- (NF-) *κ*B* via* inhibition of the phosphorylation of IkB-*α*, which are important processes in controlling inflammatory responses as well. Some of the other cancer types in which* Rhus verniciflua* Stokes has shown anti-inflammatory activities are listed in Table 1. In addition, inflammatory gene products and mechanism regulated by* Rhus verniciflua* Stokes on inflammation are listed in Table 2 specifically.

#### 2.1.4. Growth Inhibitory Effects

Many studies have indicated the growth inhibitory effects of* Rhus verniciflua* Stokes against numerous cancer cells. For instance,* Rhus verniciflua* Stokes inhibited the growth of cell proliferation in HeLa (cervical) and CT-26 (colorectal) tumor cells [7]. One study investigated the cytotoxic effects of* Rhus verniciflua* Stokes in human B, BJAB, and T lymphoma cell lines, Jurkat [8, 15].* Rhus verniciflua* Stokes was highly cytotoxic to exhibit sensitive growth inhibition in human osteosarcoma cells as well [9]. In another study,* Rhus verniciflua* Stokes exhibited a selective growth inhibition on SV40-mediated transformed embryonic hepatic cells [10].* Rhus verniciflua* Stokes showed a synergistic inhibitory effect on cell growth in gastric cancer cells at 50 *μ*g/mL [11]. Another study evaluated* Rhus verniciflua* Stokes inhibition of the clonogenic growth of small numbers of UACC-812 breast cancer cells cocultured with fibroblasts* in vitro *[16]. The extract exhibited the potent cytotoxic effects against mouse macrophage cell proliferation [17].

#### 2.1.5. Antioxidant Activity

*Rhus verniciflua* Stokes acts as a free radical scavenger in a number of* in vitro* studies (Table 1). In one study,* Rhus verniciflua* Stokes exhibited the antioxidant activity in both aqueous and lipid* in vitro* oxidation reactions using 1,1-diphenyl 2-picrylhydrazyl (DPPH) radical, site-specific Fenton-reaction deoxyribose, and a model lipid emulsion test system against hydroxyl and peroxyl radicals. In the cultured mouse brain, neurons were protected against glucose oxidase-induced hydroxyl radical in the presence of the fractionated* Rhus verniciflua* Stokes extract (e.g., 58% protection at 4.9 uM) [7]. The antioxidant activity of* Rhus verniciflua* Stokes is supported by results from other* in vitro* assays as well [25, 26]. In macrophage Raw264.7 cell and human keratinocytes model,* Rhus verniciflua* Stokes prevented the cell cytotoxicity of cells induced by H_2_O_2_, respectively, and exhibited antioxidant activities, such as DPPH, superoxide anion, and hydroxyl radical scavenging activities [28, 30].

#### 2.1.6. Antiviral Activity

*Rhus verniciflua* Stokes has been shown to inhibit the growth of viruses. In one study, the remedy inhibited the growth of fish pathogenic infectious hematopoietic necrosis virus (IHNV) and viral hemorrhagic septicemia virus (VHSV) in flounder spleen (FSP) or chinook salmon embryo- (CHSE-) 214 cells system [31].

#### 2.1.7. Neuroprotection Effects

Additionally, except for the activities discussed above,* Rhus verniciflua* Stokes exhibited numerous other neuroprotection activities by* in vitro* studies. For example, in one study,* Rhus verniciflua* Stokes protected the murine hippocampal HT22 cells against glutamate-induced neurotoxicity [32]. In another study, total extract from* Rhus verniciflua* Stokes protected dopaminergic neuronal cells in a rotenone model of Parkinson's disease (PD) [33] and against 6-hydroxydopamine- (OHDA-) induced neuronal cell death of PD. In another study,* Rhus verniciflua* Stokes protected against rotenone-induced toxicity by preventing the downregulation of brain-derived neurotrophic factor (BDNF) and glial cell line-derived neurotrophic factor (GDNF) in human dopaminergic cells, SH-SY5Y [35, 36].

#### 2.1.8. Other Activities

In addition to the activities discussed above,* Rhus verniciflua* Stokes exhibited numerous other activities by* in vitro* studies. For example, in one study,* Rhus verniciflua* Stokes showed dose-dependent inhibitory activity towards adenosine diphosphate- (ADP-), collagen-, and arachidonic acid- (AA-) induced aggregation of human platelets [37]. In another study, total extract from* Rhus verniciflua* Stokes showed Aldo-keto reductase family 1 B10 (AKR1B10), which may be responsible for detoxification of reactive aldehydes, inhibitory activity [38]. Glycoprotein isolated from* Rhus verniciflua* Stokes (RVS glycoprotein) has an inhibitory activity of T-helper type 2 (Th2) cytokines (IL-4 and -10) in bisphenol A (BPA), one of the estrogen mimic environmental hormones-stimulated primary cultured mouse lymphocytes [39].

### 2.2. Animal-Based Studies

#### 2.2.1. Antidiabetic Activity

Diabetes is a group of metabolic diseases in which a person has high blood sugar, and this disease increases the risk of long-term complications, and, therefore, it should be well regulated like inflammation.* Rhus verniciflua* Stokes has shown the potential against diabetes in many animal models. For instance, in streptozotocin- (STZ-) induced rat model,* Rhus verniciflua* Stokes exhibited a decrease in blood glucose levels and blood thiobarbituric acid reactive substance (TBARS) concentrations [44]. Another study examined the modulatory effects of* Rhus verniciflua* Stokes against hyperlipidemia in WR-1339-induced hyperlipidemic mice model [45]. This remedy decreased plasma lipid levels (total cholesterol (TC), triglyceride (TG), and low-density lipoprotein (LDL)) and inhibited the activity of 3-hydroxy-3-methylglutaryl CoA (HMG-CoA) reductase and the levels of TBARS.

#### 2.2.2. Anti-Inflammatory Effects

*Rhus verniciflua* Stokes has exhibited the potential against proinflammation in animal models as well. For instance, this remedy reduced carrageenan-induced mouse paw edema [22]. In another study, this extract showed activities on vascular permeability, leukocyte migration, and cellular immunity and reduced the incidence and severity of collagen-induced arthritis model [23].

#### 2.2.3. Antioxidative Effects

In addition to the activities discussed above,* Rhus verniciflua* Stokes exhibited antioxidative activities by* in vivo* studies. For example,* Rhus verniciflua* Stokes increased the activities of detoxicant enzymes (catalase (CAT), superoxide dismutase (SOD), and glutathione peroxidase (GPx)) in Triton WR-1339-induced hyperlipidemic mice [45].

#### 2.2.4. Hepatoprotection Effects

*Rhus verniciflua* Stokes has been shown to suppress an aflatoxin B1- (AFB1-) induced increase in serum levels of alanine aminotransferase (ALT), alkaline phosphatase (ALP), and lactate dehydrogenase (LDH), prevent malondialdehyde (MDA) formation, and block decreases in glutathione levels and SOD in mouse model [46]. In another study,* Rhus verniciflua* Stokes protected from liver damage through inhibition of radical scavenging ability [47].

#### 2.2.5. Protection from Chemical Insults

*Rhus verniciflua* Stokes has been shown to protect the normal cells, tissues, and organs against the damage caused by external insults. For instance, this remedy exhibited potential against cisplatin-induced cytotoxicity and reactive oxygen species (ROS) production in animal model [29].

#### 2.2.6. Activity against Neurodegenerative Diseases

The most common neurodegenerative disease in which* Rhus verniciflua* Stokes has shown potential is Parkinson's disease (PD). Multiple pathways including oxidative stress and mitochondrial damage have been implicated in neurodegeneration during PD. One study evaluated the neuroprotective property of* Rhus verniciflua* Stokes increased in BDNF and GDNF protein levels, which are critical for the survival and function of developing and adult neurons, learning and memory, and synaptic plasticity, in the rat brain of PD model [35].

#### 2.2.7. Other Activities

In addition to the activities indicated above,* Rhus verniciflua* Stokes modulated 12-O-tetradecanoylphorbol 13-acetate- (TPA-) induced apoptosis, cytokine production, and T/B cell proliferation in mouse splenocytes [48]. This remedy also exhibited an antifibrogenic activity by inhibition of collagen accumulation and lipid peroxidation and by the downregulation of the expression of both *α*1(I) collagen and tissue inhibitor of metalloproteinase- (TIMP-) 1 mRNA on liver fibrosis induced by carbon tetrachloride (CCl_4_) in rat model [49].

## 3. Clinical Studies with* Rhus verniciflua* Stokes

Standard modern therapies for cancer treatment include surgery, radiation, chemotherapy, hormone therapy, and palliative care. Cancer surgery still remains the foundation of treatment for cancer patients. However, patients with locally advanced disease such as gastric cancer showed high rates of locoregional or distant recurrence even after potentially curative surgery [104]. Standard chemotherapy and radiation therapy also have clinical limitation in efficacy with severe adverse effects and can act as secondary cancer promoters resulting in chemoresistant cancer cells [105, 106].* Rhus verniciflua* Stokes has been tested for its potential in human subjects, with about a dozen studies completed to date. Most of these studies have indicated the safety and efficacy of* Rhus verniciflua* Stokes. The most promising effect of* Rhus verniciflua* Stokes has been reported against cancer (Table 4). One study demonstrated that* Rhus verniciflua* Stokes can be administered safely to patients with metastatic colorectal cancer (mCRC) at doses of 450 mg of* Rhus verniciflua* Stokes that was prescribed [50]. Ten among 36 patients were alive after treatment with 2.7 months (95% confidence interval, 1.9–3.5) median administration period, 10.9 months (95% confidence interval, 5.6–16.1) median overall survival (OS), and 44.4% 1-year survival rate. Although the effects of* Rhus verniciflua* Stokes continued for several months, hematologic toxicity was not observed and minor adverse effects-mild pruritus and dyspepsia was reported in only 2 of the 36 patients. However, a large scale study is required to further confirm the efficacy and safety of* Rhus verniciflua* Stokes in mCRC patients and this study has the limitation that 44.4% of patients have chosen* Rhus verniciflua* Stokes as the complementary therapy with conventional treatment including chemotherapy or radiotherapy. In another study,* Rhus verniciflua* Stokes treatment was well tolerated in advanced pancreatic cancer patients for whom orthodox therapy is unavailable and might prolong overall survival either alone or in combination with chemotherapy [54]. Three out of 42 patients were alive with 3.86 months (95% confidence interval 2.52–5.20) mean administration period, 7.87 months (95% confidence interval 5.14–10.59) median overall survival, and 26.2% 1-year survival rate. Hematologic toxicity related to only* Rhus verniciflua* Stokes oral administration was not observed; minor nonhematologic adverse reactions were reported such as mild dyspepsia and pruritus in each patient with toxicity grade 1 pruritus and grade 2 pruritus, respectively. Gemcitabine has emerged as the standard chemotherapy for advanced pancreatic cancer [107]. Many clinical trials have been performed to improve survival by comparing gemcitabine with other agents, either alone or in combination with gemcitabine [108, 109]. In this study, among the patients treated with* Rhus verniciflua* Stokes and concurrent chemotherapy, 19.0% of patients with grade 3 or 4 toxicity were required to discontinue gemcitabine treatment, all patients were not observed a synergistic effect of gemcitabine and* Rhus verniciflua *Stokes compared to alone. Although* Rhus verniciflua* Stokes oral administration was very tolerable, no specific drug interactions between* Rhus verniciflua* Stokes and chemotherapy agents were noted, but additional randomized and well-controlled clinical trials with larger number of patients are necessary to confirm its efficacy and safety in the treatment of pancreatic cancer.

In addition to the clinical research indicated above,* Rhus verniciflua* Stokes has also been reported to have anticancer activity in various kinds of cancers including gastric, liver, lung, renal, and pulmonary. In the 82-year-old female gastric cancer patient case, orally administered* Rhus verniciflua* Stokes decreased the polypoid mass at the mid body and a slight decrease in the flat elevated lesion at the prepyloric antrum at 5 months after starting daily therapy with 900 mg [51]. Another 62-year-old Korean male patient with recurrent hepatocellular carcinoma after liver transplantation refractory to doxorubicin exhibited shrinkage of the lung metastasis, nonhematologic toxicity at 5 months after receiving* Rhus verniciflua *Stokes 3 times a day with 450 mg being orally administered [52]. Moreover, two case studies were reported against renal cancer in 2010 as well [53]. Of these, one case* Rhus verniciflua *Stokes three times in a day with 450 mg capsules for 4 months exhibited a complete response in all pulmonary metastases including resolution of right pulmonary artery thrombosis, and the other case showed reduction in the size of the metastatic masses in both adrenal glands at 9 months after receiving* Rhus verniciflua *Stokes 3 times a day with 450 mg capsules being orally administrated. Another study reported the case of a 52-year-old female who had been diagnosed with pulmonary adenocarcinoma with malignant pleural nodules [55]. The patient maintained good performance status with European Cooperative Oncology Group (ECOG) performance status of 0 for 2 years after daily therapy (1,350 mg of orally administered* Rhus verniciflua *Stokes remedy without orthodox therapies) and no significant adverse effects. Orally administered* Rhus verniciflua *Stokes showed an obvious cytostatic effect and could increase the quantity of survival in pulmonary adenocarcinoma; however, further studies with larger populations are required to confirm the claims of the study.

## 4. Main Compounds from* Rhus verniciflua* Stokes

### 4.1. Chemical Composition of* Rhus verniciflua* Stokes

**Figure 2:**
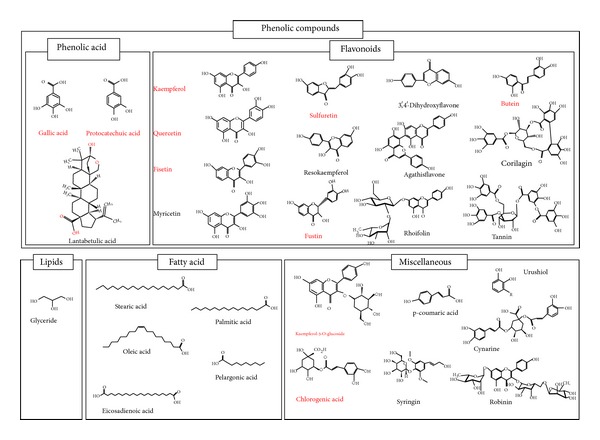
Molecular structure of common constituents of* Rhus verniciflua* Stokes.

**Figure 3:**
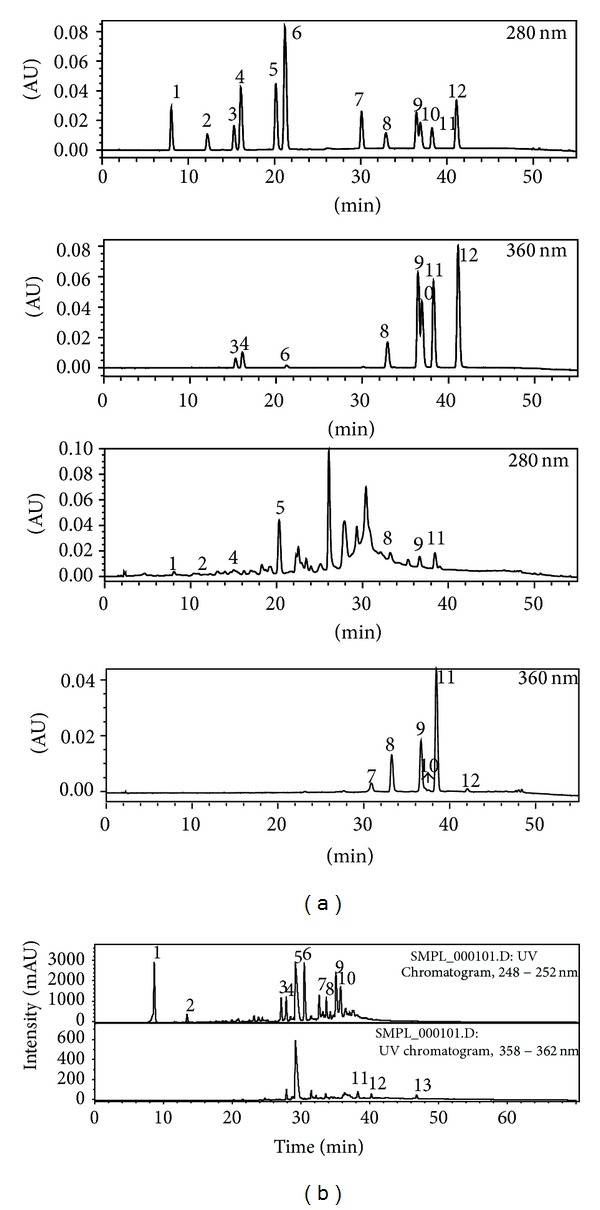
High-performance liquid chromatography (HPLC) and liquid chromatography-mass spectrometry (LC-MS) analysis of constituents identified from* Rhus verniciflua* Stokes. (a) HPLC chromatogram of standard compounds (upper) and purified* Rhus verniciflua* Stokes extract (lower) at 280 nm and 360 nm: 1: protocatechuic acid, 2:* p*-hydroxybenzoic acid, 3: caffeic acid, 4: chlorogenic acid, 5:* p*-coumaric acid, 6: phloretin-2-*O*-glucoside, 7: fustin, 8: kaempferol-3-*O*-glucoside, 9: sulfuretin, 10: quercetin, 11: butein, and 12: kaempferol. (b) LC-MS chromatogram of phenolic-rich EtOAC fraction from* Rhus verniciflua* Stokes extract: 1: gallic acid, 2: protocatechuic acid, 3–9: 8 unknown compounds, 11: fisetin, 12: sulfuretin, and 13: butein.

*Rhus verniciflua* Stokes is chemically diverse in composition. To date, around 40 compounds, primarily phenolic acids and flavonoids, have been identified from this remedy (Figure 2) [14, 18, 110]. Of these compounds, 3 are phenolic acid, 4 flavonols, 4 flavanonols, 3 flavones, 1 chalconoid, and 2 tannins. The most common constituents present in* Rhus verniciflua* Stokes are butein, which is a chalconoid having antibacterial, antifungal, antitumor, and anti-inflammatory properties, quercetin, which is a flavonol having antiviral, antiasthma, anticancer, antiprostatitis, and anti-inflammatory properties, and sulfuretin, which is a flavanonol possessing antioxidative, antidiabetes, anticancer, antiviral, and anti-inflammatory properties. The chemical structure and high-pressure liquid chromatography (HPLC) and liquid chromatography-mass spectrometry (LC-MS) analysis of some other compounds identified from* Rhus verniciflua* Stokes are shown in Figures 2 and 3 [14, 18].

### 4.2. Biological Activities of Main Compounds from* Rhus verniciflua* Stokes

Many researches have been studied to identify the clinically active ingredient from* Rhus verniciflua* Stokes like aspirin which was first discovered from the bark of the willow tree in 1763 by Edward Stone of Wadham College, Oxford University. Recently flavonoids from* Rhus verniciflua* Stokes have been found to have various biological activities, including antiproliferative, anti-inflammatory, and apoptotic activities in human cancer cell lines and* in vivo* model. For example, butein from* Rhus verniciflua* Stokes inhibited clonogenic growth of human breast cancer cells cocultured with fibroblasts [16], human colon adenocarcinoma cell proliferation [111], and prostate tumor growth* in vitro* and* in vivo* [59]. Moreover, this chalcone exhibited inhibition of NF-*κ*B activation and infiltration reduction of inflammatory cells and apoptosis after spinal cord injury in animal model [60]. In another study, this tetrahydroxychalcone protected the murine hippocampal HT22 cells against glutamate-induced neurotoxicity, attenuated reactive oxygen species (ROS) generations through preserving the activities of SOD, GR, and GSH-Px, showed inhibitory effects on LPS-induced NO production, and suppressed the expression of both iNOS and COX-2 in BV2 cells [32]. Other main compounds, kaempferol, quercetin, and fisetin, have similar biological activities, including antiproliferative, anti-inflammatory, antioxidative, and apoptotic activities in human cancer cell lines and* in vivo* model. Their activities could come from a similar structure base. Some of the other constituents in which* Rhus verniciflua* Stokes has shown biological activities are listed in Table 5.

### 4.3. Biological Activities of Urushiol from* Rhus verniciflua* Stokes

Urushiol is an allergen found in Anacardiaceae family, especially* Toxicodendron* spp. In sensitive individuals, urushiol can cause an allergic response [112]. Many researchers concerned about this compound could induce allergy during disease treatment with* Rhus verniciflua* Stokes. All clinical studies indicated in Section 3 used an urushiol-free extract of* Rhus verniciflua* Stokes and the treatment of this remedy has no severe adverse effect. However, this is still controversial because more detailed confirmative works on safety will be also required to use this remedy clinically in the world.

## 5. Conclusion

Whereas modern medicine has developed chemotherapy drugs for single-targeted agents, the traditional medicine is for multitargeted agents. The usage of entire or part extraction from a plant probably alleviates the adverse effects and drug resistance which are major problems in modern medicine [113].* Rhus verniciflua* Stokes is an Asian tree species of genus* Toxicodendron,* which belongs to Anacardiaceae family. Traditionally, the remedy has been used for enormous purposes since ancient times. Modern science has provided the molecular basis for the properties of* Rhus verniciflua* Stokes against human diseases using* in vitro* and* in vivo* model, and the existing human studies have provided a logical basis for further investigation of this remedy for the prevention and treatment of human diseases, especially cancer. In addition to this accomplishment, clinical studies have demonstrated the safety and efficacy of* Rhus verniciflua* Stokes in human subjects. The absence of any significant adverse effect associated with this remedy has made it superior to others. However, future studies should focus on employing larger, high-quality clinical trials, demonstrating its efficacy in terms of cancer patients' survival and quality of life, and measuring cost-effectiveness in clinical practice. Additionally,* Rhus verniciflua* Stokes is a rich source of numerous biologically active constituents such as flavonoids, phenolic compounds which have anti-inflammatory activity, and chalconoids, which have antibacterial, antifungal, antitumor, and anti-inflammatory properties.

## Figures and Tables

**Table 1:** Biological activities of *Rhus verniciflua* Stokes as shown in *in vitro* studies.

**Antibacterial**	
Exhibited activity against *H. pylori* [6].	
**Anticancer**	
(i) Exhibited 70% cell death in HeLa and CT-26 tumor cell lines at a minimum concentration of 2.48 *μ*M [7].	
(ii) Increased DNA fragmentation on the human B and T lymphoma cell lines, BJAB and Jurkat [8].	
(iii) Exhibited apoptosis *via* caspase-8/PARP cleavage pathway in human osteosarcoma cells [9].	
(iv) Exhibited apoptosis induction on SV40-mediated transformed embryonic hepatic cells [10].	
(v) Induced apoptosis through an intrinsic pathway in gastric cancer cell lines [11].	
(vi) Exhibited caspase-independent death of human osteosarcoma cells *via* p53-mediated mitochondrial stress and nuclear translocation of AIF and endonuclease G [12].	
(vii) Enhanced mitochondrial mediated apoptosis by inhibition of the PI3K-Akt/PKB survival pathway in gastric cancer cell lines [13].	
(viii) Exhibited potential organ-specific anticancer activity [14].	
**Antigrowth activity**	
(i) Inhibited cell proliferation in cultured HeLa and CT-26 tumor cells [7].	
(ii) Inhibited the growth of human B, BJAB, and T lymphoma cell lines, Jurkat [8, 15].	
(iii) Exhibited sensitive growth inhibition in human osteosarcoma cells [9].	
(iv) Exhibited a selective growth inhibition on SV40-mediated transformed embryonic hepatic cells [10].	
(v) Exhibited a synergistic inhibitory effect on cell growth in gastric cancer cells at 50 *μ*g/mL [11].	
(vi) Inhibited the clonogenic growth of small numbers of UACC-812 breast cancer cells cocultured with fibroblasts *in vitro* [16].	
(vii) Suppressed mouse macrophage cell proliferation [17].	
**Anti-inflammatory**	
(i) Suppressed proinflammatory mediators NO, PGE_2_, and TNF-*α* *via* inhibition of NF-*κ*B and JNK pathway in LPS-induced RAW 264.7 macrophages [18].	
(ii) Inhibited ROS production and PKC-*α* translocation, downregulated the expression of NF-*κ*B and AP-1, and inhibited the levels of iNOS and COX-2 expression [19].	
(iii) Inhibited the expressions of TNF-*α*, IL-6, and IL-8 on human mast cells with treatment with PMA and A23187 [20].	
(iv) Inhibited LPS-induced NO, PGE_2_, TNF-*α*, and IL-1*β* production *via* the induction of HO-1 expression in murine macrophages [21].	
(v) Suppressed NOS *via* the ERK and Akt signaling pathways [22].	
(vi) Suppressed iNOS and COX2 mRNA expression induced by LPS and decreased intracellular ROS levels induced by LPS [17].	
(vii) Inhibited inflammation-related cytokines and angiogenic factor in rheumatoid arthritic fibroblast-like synovial cells [23].	
(viii) Suppressed 2,4-DNFB-induced allergic contact dermatitis [24].	
**Antioxidative**	
(i) Exhibited the inhibition of hydroxyl radical-mediated degradation by iron ion chelation [25].	
(ii) Exhibited the inhibition of linoleic acid oxidation, protected human LDL from oxidative modification, and protected against plasmid DNA strand breakage induced by peroxyl free radicals [26].	
(iii) Exhibited against hydroxyl and peroxyl radicals in *in vitro* assays [7].	
(iv) Inhibited activities of NF-*κ*B and AP-1 induced by G/GO [27].	
(v) Reduced intracellular ROS formation caused by H_2_O_2_, reduced TBARS formation, and attenuated catalase depletion at concentration of 100 *μ*/mL [28].	
(vi) Prevented cisplatin-induced ROS release against MDCK-I cells [29].	
(vii) Protected human keratinocytes against oxidative stress caused by H_2_O_2_ [30].	
**Antiviral**	
Exhibited antiviral activity against fish pathogenic IHNV and VHSV [31].	
**Neuroprotection**	
(i) Protected the murine hippocampal HT22 cells against glutamate-induced neurotoxicity [32].	
(ii) Protected dopaminergic neuronal cells in a rotenone model of PD [33].	
(iii) Protected against 6-OHDA-induced neuronal cell death of PD [34].	
(iv) Protected against rotenone-induced toxicity by preventing the downregulation of BDNF and GDNF in human dopaminergic cells, SH-SY5Y [35, 36].	
**Other activities**	
(i) Inhibited platelet aggregation *via* inhibition of receptor expression on platelet membranes, including glycoprotein IIb/IIIa (CD41), GPIIb/IIIa-like expression (PAC-1), and P-selectin (CD62), and intracellular calcium mobilization responses and decreased platelet activation were observed for the isomaltol- and pentagalloyl glucose-treated platelets [37].	
(ii) Exhibited anti-AKR1B10 activity at 1 *μ*M with an IC_50_ value of 1.47 *μ*M [38].	
(iii) Suppressed IL-4 and -10 in BPA-stimulated primary cultured mouse lymphocytes [39].	

AIF: apoptosis-inducing factor; AKR1B10: Aldo-keto reductase family 1 B10; AP-1: activator protein-1; BDNF: brain-derived neurotrophic factor; BPA: bisphenol A; COX-2: cyclooxygenase-2; DNA: deoxyribonucleic acid; 2,4-DNFB: 2,4-dinitrofluorobenzene; GDNF: glial cell line-derived neurotrophic factor; G/GO: glucose/glucose oxidase; HO: heme oxygenase; *H*. *pylori: Helicobacter pylori*; IC_50_: the half maximal inhibitory concentration; IHNV: infectious hematopoietic necrosis virus; IL: interleukin; iNOS: inducible nitric oxide synthase; JNK: c-Jun NH(2)-terminal kinase; LDL: low-density lipoprotein; LPS: lipopolysaccharide; NF-*κ*B: nuclear factor kappa B; NO: nitric oxide; NOS: nitric oxide synthase; OHDA: hydroxydopamine; PARP: poly (ADP-ribose) polymerase; PD: Parkinson's disease; PGE_2_: prostaglandin E_2_; PI3K: Phosphatidylinositide 3-kinases; PKB: protein kinase B; PKC: protein kinase C; PMA: phorbol 12-myristate 13-acetate; ROS: reactive oxygen species; SV40: Simian virus 40; TBARS: thiobarbituric acid reactive substance; TNF: tumor necrosis factor; VHSV: viral hemorrhagic septicemia virus.

**Table 2:** Inflammatory gene products and mechanism regulated by *Rhus verniciflua* Stokes.

Model	Inducer	Mechanism (target genes)	[References]
Cell lines			
Macrophage	LPS	Inhibited NO, PGE_2_, and TNF-*α* production Reduced NF-*κ*B activity Suppressed iNOS and COX-2 protein expression *via* inactivation of JNK1/2 MAPK kinase pathway	[18]
Macrophage	LPS	Inhibited ROS production PKC-*α* translocation Downregulated the expression of NF-*κ*B and AP-1 Inhibited the levels of iNOS and COX-2 expression	[19]
Macrophage	LPS	Reduced iNOS at the transcriptional level Downregulated iNOS protein expression *via* the ERK and Akt pathway	[22]
HMC-1	PMA, A23187	Inhibited the expressions of TNF-*α*, IL-6, and IL-8 Suppressed the phosphorylation of ERK and p38 but not JNK Inhibited the nuclear translocation of NF-*κ*B *via* inhibition of the phosphorylation of I*κ*B-*α*	[20]
FLS	IL-1*β*	Decreased TNF-*α*, IL-6, IL-8, MCP-1, and VEGF Decreased the expression of VEGF *via* the phosphorylation of p38 MAPK pathway	[23]
Macrophage	LPS	Inhibited NO, PGE_2_, TNF-*α*, and IL-1*β* production via the induction of HO-1 expression	[21]
Animals			
Mouse	Carrageenan	Reduced paw edema	[22]
Mouse	Acetic acid	Decreased peritoneal capillary permeability	
	CMC-Na	Significantly decreased leukocytes migration in peritoneal cavity	
	Oxazolone	Inhibited ear thickness (DTH)	
	Collagen	Reduced the incidence and severity of CIA	[21]
Mouse	2,4-DNFB	Reduced ear swelling, hyperplasia of ear tissue	
		Increased vascular permeability	
		Decreased numbers of infiltrated mast cells	[24]

AP-1: activator protein-1; CIA: collagen-induced arthritis; COX-2: cyclooxygenase-2; 2,4-DNFB: 2,4-dinitrofluorobenzene; DTH: delayed type hypersensitivity; FLS: rheumatoid arthritic fibroblast-like synovial cells; HMC-1: human mast cells; HO: heme oxygenase; IL: interleukin; iNOS: inducible nitric oxide synthase; JNK: c-Jun NH(2)-terminal kinase; LPS: lipopolysaccharide; MCP-1: monocyte chemoattractant protein; NF-*κ*B: nuclear factor kappa B; NO: nitric oxide; NOS: nitric oxide synthase; oxazolone: 4-ethoxymethylene-2-phenyloxazolone; PGE_2_: prostaglandin E_2_; PKC: protein kinase C; PMA: phorbol 12-myristate 13-acetate; ROS: reactive oxygen species; TNF: tumor necrosis factor; VEGF: vascular endothelial growth factor.

**Table 3:** Biological activities of *Rhus verniciflua* Stokes as shown in *in vivo* studies.

Model	Effect
Antidiabetic	
Rat	Exhibited a decrease in blood glucose levels and blood TBARS concentrations in STZ-induced diabetic rats [44].
Mouse	Decreased in plasma lipid levels (TC, TG, and LDL) and inhibited the activity of HMG-CoA reductase and the levels of TBARS in Triton WR-1339-induced hyperlipidemic mice [45].
Anti-inflammatory	
Mouse	Reduced carrageenan-induced mouse paw edema [22].
Mouse	Exhibited activities on vascular permeability, leukocyte migration, and cellular immunity and reduced the incidence and severity of collagen-induced arthritis model [29].
Antioxidative	
Mouse	Increased the activities of detoxicant enzymes (CAT, SOD, and GPx) in Triton WR-1339-induced hyperlipidemic mice [45].
Hepatoprotection	
Mouse	Suppressed an AFB1-induced increase in serum levels of ALT, ALP, and LDH, prevented MDA formation, and blocked decreases in glutathione levels and SOD [46].
Mouse	Protected from liver damage through inhibited radical scavenging ability, enhanced the activities of antioxidant enzymes, increased the NO production, and decreased the NF-*κ*B and AP-1 activations [47].
Neurodegenerative diseases	
Rat	Increased in BDNF and GDNF protein levels in the rat brain [35].
Protection from chemical insults	
Mouse	Exhibited potential against cisplatin-induced cytotoxicity and ROS production [29].
Other activities	
Mouse	Modulated TPA-induced apoptosis, cytokine production, and T/B cell proliferation in splenocytes [48].
Rat	Exhibited an antifibrogenic activity by inhibition of collagen accumulation and lipid peroxidation and by downregulation of the expression of both *α* 1(I) collagen and TIMP-1 mRNA on liver fibrosis induced by CCl_4_ [49].

AFB-1: aflatoxin B-1; ALP: alkaline phosphatase; ALT: alanine aminotransferase; AP-1: activator protein-1; BDNF: brain-derived neurotrophic factor; CAT: catalase; CCl_4_: carbon tetrachloride; GDNF: glial cell line-derived neurotrophic factor; GPx: glutathione peroxidase; HMG-CoA: 3-hydroxy-3-methylglutaryl CoA; LDH: lactate dehydrogenase; LDL: low-density lipoprotein; MDA: malondialdehyde; NF-*κ*B: nuclear factor kappa B; NO: nitric oxide; ROS: reactive oxygen species; SOD: superoxide dismutase; STZ: streptozotocin; TBARS: thiobarbituric acid reactive substance; TC: total cholesterol, TG: triglyceride; TIMP-1: tissue inhibitor of metalloproteinases 1; TPA: 12-O-tetradecanoylphorbol 13-acetate.

**Table 4:** Biological activities of *Rhus verniciflua* Stokes as shown in clinical studies.

Type	Effect
Anticancer	
Colon	Ten among 36 patients were alive after treatment with 2.7 months (95% confidence interval, 1.9–3.5) median administration period, 10.9 months (95% confidence interval, 5.6–16.1) median overall survival, and 44.4% 1-year survival rate [50].

Gastric	Case study: decreased the polypoid mass at the mid body and a slight decrease in the flat elevated lesion at the prepyloric antrum at 5 months after starting daily therapy with 900 mg of orally administered [51].

Liver	Case study: patient with recurrent hepatocellular carcinoma after liver transplantation refractory to doxorubicin exhibited shrinkage of the lung metastasis, nonhematologic toxicity at 5 months after receiving 3 times in a day with 450 mg orally administered [52].

Renal	(i) Case study I: exhibited a complete response in all pulmonary metastases including resolution of right pulmonary artery thrombosis when given at 450 mg capsules with three times a day for 4 months [53]. (ii) Case study II: showed reduction in the size of the metastatic masses in both adrenal glands at 9 months after receiving 3 times in a day with 450 mg capsules orally [53].

Pancreatic	Three among 42 patients were alive with 3.86 months (95% confidence interval 2.52–5.20) mean administration period, 7.87 months (95% confidence interval 5.14–10.59) median overall survival, and 26.2% 1-year survival rate [54].

Pulmonary	(i) Case study: maintained good performance status with ECOG performance status of 0 for 2 years after treating daily therapy with 1,350 mg of orally administered remedy without orthodox therapies and no significant adverse effects [55]. (ii) Reviewed the medical records of 33 patients with advanced NSCLC, who treated this remedy after completion of four or six cycles of induction chemotherapy for 6 years. A 6- and 12-month PFS rate was 40.6% and 12.9%, respectively. The DCR was 93.9% and the median OS was 34.8 months with 12, 24, and 36 months of overall survival rates which were 84.2%, 76.7%, and 49.9%, respectively. No hematologic toxicity, nephrotoxicity, or hepatotoxicity [56].

DCR: disease control rate; ECOG: European Cooperative Oncology Group; NSCLC: non-small-cell lung carcinoma; OS: overall survival; PFS: progression-free survival.

**Table 5:** Selected biological activities of selected main compounds from *Rhus verniciflua* Stokes.

Type	Effect
Butein	(i) Exhibited aldose reductase and advanced glycation end-products inhibition [57]. (ii) Protected pancreatic beta cells (INS-1 cells) against cytokine-induced toxicity mediated by inhibition of NO formation at concentrations of 15–30 *μ*M [58]. (iii) Protected the murine hippocampal HT22 cells against glutamate-induced neurotoxicity, attenuated ROS generations through preserving the activities of SOD, GR, and GSH-Px [32]. (iv) Inhibited clonogenic growth of human breast cancer cells cocultured with fibroblasts. (v) Inhibited prostate tumor growth *in vitro* and *in vivo* [59]. (vi) Inhibited NF-*κ*B activation and reduces infiltration of inflammatory cells and apoptosis after spinal cord injury in rats [60].

Fisetin	(i) Exhibited antibacterial effect [61]. (ii) Protected cultured rat liver epithelial-like cells against AFB-1-induced cytotoxicity and inhibited the binding of [3] AFB-1 to cellular DNA [62]. (iii) Exhibited a predilection to inhibit histamine release stimulated by IgE-dependent ligands (antigen, anti-IgE, and con A) [63]. (iv) Inhibited TPA-caused epidermal ornithine decarboxylase induction and tumor promotion in relation to lipoxygenase inhibition [64]. (v) Inhibited PKC, almost 100% inhibition at a concentration of 100 micro-M from rat brain [65]. (vi) Suppressed mutagenesis in *Salmonella typhimurium* strain TA100 NR induced by direct-acting carcinogen N-methyl-N′-nitro-N-nitrosoguanidine [66]. (vii) Showed topoisomerase II dependent DNA cleavage activity [67]. (viii) Inhibited platelet aggregation [68]. Attenuated NO production in C6 astrocyte cell [69]. (ix) Blocked glucose uptake in myelocytic U937 cells [70]. (x) Inhibited corneal neovascularization; corneal blood vessels were induced by intrastromal implantation of pellets containing bFGF [71]. (xi) Inhibited the proliferation of HSC-T6 cells, hepatic stellate cells stimulated by serum, MCM, and PDGF [72]. (xii) Inducted apoptosis through activation of caspase-3 cascade and alternative expression of p21 protein in hepatocellular carcinoma cells SK-HEP-1 [73]. (xiii) Exhibited antiviral activities against IHNV and VHSV [31]. (xiv) Inhibited IL-4 and IL-13 synthesis and production by allergen- or anti-IgE-antibody-stimulated basophils [74]. (xv) Protected against hepatosteatosis in mice by inhibiting miR-378 [75]. (xvi) Protected bone by repressing NF-*κ*B and MKP-1-dependent signaling pathways in osteoclasts [76]. (xvii) Enhanced behavioral performances and attenuated reactive gliosis and inflammation during aluminum chloride-induced neurotoxicity [77]. (xviii) Recuperated antioxidant status and protected hepatocellular ultrastructure from hyperglycemia mediated oxidative stress in STZ-induced rats diabetes model [78].

Kaempferol	(i) Inhibited estrogen binding to serum alpha-fetoprotein AFP in fetal or neonatal rats [79]. (ii) Showed antioxidative activity against metal-induced lipid peroxidation [80]. (iii) Suppressed TNF-*α*-stimulated E-selectin expression on HUVECs [81]. (iv) Exhibited high inhibitory potencies for the 20alpha-HSD activity on liver cytosol of male mice [82]. (v) Inhibited IgE or PMACI-mediated histamine release in RBL-2H3 cells and inhibited elevation of intracellular calcium [83].

Fustin	(i) Exerted inhibition of cell proliferation on Molt-4 cell and normal lymphocyte and enhanced IL-2 level [84]. (ii) Suppressed 6-OHDA-induced cell death, blocked 6-OHDA-induced increases in ROS, [Ca(2+)](i), Bax/Bcl-2 ratio, caspase-3 activity, and p38 phosphorylation [34]. (iii) Attenuated Abeta(1–42)-impaired learning [85]. (iv) Displayed antiviral activities against IHNV and VHSV [31].

Sulfuretin	(i) Exhibited potent antioxidants in a DPPH free radical scavenging assay [86]. (ii) Exhibited aldose reductase and advanced glycation end-products inhibition [57]. (iii) Inhibited iNOS and COX-2 protein and mRNA expression and reduced iNOS-derived NO, COX-derived PGE_2_, TNF-*α*, and IL-1*β* production in LPS-stimulated RAW264.7 and murine peritoneal macrophages [21]. (iv) Reduced cytokine (IL-1 *β*- and IFN-*γ*-) induced NF-*κ*B activation, iNOS expression, and NO production in rat insulinoma RINm5F cells, and prevented STZ-induced hyperglycemia and hypoinsulinemia by suppression of NF-*κ*B activation [87]. (v) Inhibited NF-*κ*B pathway, suppressed the production of various cytokines in bronchoalveolar fluid and mucin production, and prevented the development of airway hyperresponsiveness on an ovalbumin-induced airway inflammation model in mice [88]. (vi) Induced apoptosis through activation of Fas, caspase-8, and the mitochondrial death pathway in HL-60 human leukemia cells [89]. (vii) Blocked NF-*κ*B pathway in rheumatoid joints and reduced inflammatory responses and joint destruction [90]. (viii) Inhibited TPA-induced NF-*κ*B activation, MMP-9 expression, and cell invasion in MCF-7 cells [91]. (ix)Induced miR-30C, downregulated cyclins D1 and D2, and triggered cell death in human cancer cell [92].

Quercetin	(i) Induced apoptosis in colorectal tumor cells *via* EGF receptor signaling [93]. (ii) Showed antioxidative activity against metal-induced lipid peroxidation [80]. (iii) Induced glutathione S-transferase and increased the resistance of cells to hydrogen peroxide [94]. (iv) Inhibited the proliferation of HSC-T6 cells and hepatic stellate cells stimulated by serum, MCM, and PDGF [72]. (v) Inhibited the antigen-IgE-mediated TNF-*α* and IL-4 production from RBL-2H3 [95]. (vi) Decreased the amount of myelin phagocytosed by a macrophage cell line [96]. (vii) Induced apoptosis through the activation of caspase-3 and caspase-8 in human leukemia U937 cells [97]. (viii) Inhibited Abeta fibril formation on neuronal HT22 murine neuroblastoma cells [98]. (ix) Inhibited prokaryotic SssI DNMT- and human DNMT1-mediated DNA methylation [99]. (x) Showed inhibitory effects against HSV-1 [100]. (xi) Exhibited inhibitory potencies for the 20alpha-HSD activity using liver cytosol of male mice [82]. (xii) Inhibited EGF-induced cell transformation of mouse epidermal JB6 Cl 41 cells [101]. (xiii) Inhibited tumor invasion *via* suppressing PKC Δ/ERK/AP-1-dependent MMP-9 activation in breast carcinoma cells [102]. (xiv) Inhibited the cell proliferation induced by 17-beta-estradiol in the E-screen assay (the evaluation of antiestrogenicity) [103].

20alpha-HSD: 20alpha-hydroxysteroid dehydrogenase; 6-OHDA: 6-hydroxydopamine; AFB1: aflatoxin B1; Abeta: amyloid-beta; AP-1: activator protein-1; COX-2: cyclooxygenase-2; DCR: disease control rate; DNA: deoxyribonucleic acid; DNMT: DNA methyltransferase; DPPH: 1,1-diphenyl-2-picrylhydrazyl; ECOG: European Cooperative Oncology Group; EGF: epidermal growth factor; bFGF: basic fibroblast growth factor; ERK: extracellular signal-regulated kinases; GHB: gamma-hydroxybutyrate; GR: glutathione reductase; GSH: glutathione; GSH-Px: glutathione peroxidase; HO: heme oxygenase; HSV-1: herpes simplex virus type 1; IkB-alpha: inhibitory kappa B-alpha; IHNV: infectious hematopoietic necrosis virus; IKK beta: I kappaB kinase beta; IL-1 beta: interleukin-1 beta; iNOS: inducible nitric oxide synthase; LPS: lipopolysaccharide; MCM: macrophage conditioned medium; MKP-1: mitogen-activated protein kinase phosphatase-1; MMP: matrix metalloproteinase; NF-kappaB: nuclear factor-kappaB; NO: nitric oxide; nrf2: nuclear factor E2-related factor 2; NSCLC: non-small-cell lung carcinoma; OS: overall survival; PDGF: platelet-derived growth factor; PFS: progression-free survival; PGE2: prostaglandin E2; PKC: protein kinase C; PMACI: phorbol-12-myristate 13-acetate and calcium ionophore A23187; ROS: reactive oxygen species; SOD: superoxide dismutase; STZ: streptozotocin; TNF-alpha: tumor necrosis factor-alpha; TPA: 12-O-tetradecanoylphorbol-13-acetate; VHSV: viral hemorrhagic septicemia virus.

## References

[1] Aggarwal BB, Gupta SC, Park B, Yadav VR, Kim JH (2011). Chronic diseases caused by chronic inflammation require chronic treatment: the anti-inflammatory lifestyle. *Inflammation, Lifestyle and Chronic Diseases: The Silent Link*.

[2] Aggarwal BB, Ichikawa H, Garodia P (2006). From traditional Ayurvedic medicine to modern medicine: identification of therapeutic targets for suppression of inflammation and cancer. *Expert Opinion on Therapeutic Targets*.

[3] Hsiao WLW, Liu L (2010). The role of traditional Chinese herbal medicines in cancer therapy—from TCM theory to mechanistic insights. *Planta Medica*.

[4] Network UARSGRI Toxicodendron vernicifluum information from NPGS/GRIN.

[5] Yoo HT, Roh JR (1977). *Compendium of Prescriptions From the Countryside (Hyangyakjipseongbang)*.

[6] Suk KT, Baik SK, Kim HS (2011). Antibacterial effects of the urushiol component in the sap of the lacquer tree (*Rhus verniciflua* stokes) on Helicobacter pylori. *Helicobacter*.

[7] Kitts DD, Lim K-T (2001). Antitumorigenic and cytotoxic properties of an ethanol extract derived from *Rhus verniciflua* stokes (RVS). *Journal of Toxicology and Environmental Health A*.

[8] Lee J-C, Kim J, Jang Y-S (2003). Ethanol-eluted extract of *Rhus verniciflua* stokes inhibits cell growth and induces apoptosis in human lymphoma cells. *Journal of Biochemistry and Molecular Biology*.

[9] Jang H-S, Kook S-H, Son Y-O (2005). Flavonoids purified from *Rhus verniciflua* Stokes actively inhibit cell growth and induce apoptosis in human osteosarcoma cells. *Biochimica et Biophysica Acta—General Subjects*.

[10] Son Y-O, Lee K-Y, Lee J-C (2005). Selective antiproliferative and apoptotic effects of flavonoids purified from *Rhus verniciflua* Stokes on normal versus transformed hepatic cell lines. *Toxicology Letters*.

[11] Kim JH, Kim H-P, Jung C-H (2006). Inhibition of cell cycle progression via p27Kip1 upregulation and apoptosis induction by an ethanol extract of *Rhus verniciflua* Stokes in AGS gastric cancer cells. *International Journal of Molecular Medicine*.

[12] Kook S-H, Son Y-O, Chung S-W (2007). Caspase-independent death of human osteosarcoma cells by flavonoids is driven by p53-mediated mitochondrial stress and nuclear translocation of AIF and endonuclease G. *Apoptosis*.

[13] Kim JH, Go HY, Jin DH (2008). Inhibition of the PI3K-Akt/PKB survival pathway enhanced an ethanol extract of *Rhus verniciflua* Stokes-induced apoptosis via a mitochondrial pathway in AGS gastric cancer cell lines. *Cancer Letters*.

[14] Kim JH, Jung CH, Jang B-H (2009). Selective cytotoxic effects on human cancer cell lines of phenolic-rich ethyl-acetate fraction from *Rhus verniciflua* stokes. *The American Journal of Chinese Medicine*.

[15] Lee J-C, Lee K-Y, Kim J (2004). Extract from *Rhus verniciflua* Stokes is capable of inhibiting the growth of human lymphoma cells. *Food and Chemical Toxicology*.

[16] Samoszuk M, Tan J, Chorn G (2005). The chalcone butein from *Rhus verniciflua* Stokes inhibits clonogenic growth of human breast cancer cells co-cultured with fibroblasts. *BMC Complementary and Alternative Medicine*.

[17] Choi HS, Seo HS, Kim SR (2014). Antiinflammatory and antiproliferative effects of *Rhus verniciflua* Stokes in RAW264. 7 cells. *Molecular Medicine Reports*.

[18] Jung CH, Kim JH, Hong MH (2007). Phenolic-rich fraction from *Rhus verniciflua* Stokes (RVS) suppress inflammatory response via NF-*κ*B and JNK pathway in lipopolysaccharide-induced RAW 264.7 macrophages. *Journal of Ethnopharmacology*.

[19] Oh P-S, Lee S-J, Lim K-T (2007). Glycoprotein isolated from *Rhus verniciflua* STOKES inhibits inflammation-related protein and nitric oxide production in LPS-stimulated RAW 264.7 cells. *Biological and Pharmaceutical Bulletin*.

[20] Hong MH, Kim J-H, Lee SY (2010). Early antiallergic inflammatory effects of *Rhus verniciflua* stokes on human mast cells. *Phytotherapy Research*.

[21] Lee D-S, Jeong G-S, Li B, Park H, Kim Y-C (2010). Anti-inflammatory effects of sulfuretin from *Rhus verniciflua* Stokes via the induction of heme oxygenase-1 expression in murine macrophages. *International Immunopharmacology*.

[22] Jung CH, Kim J-H, Kim JH (2011). Anti-inflammatory effect of *Rhus verniviflua* Stokes by suppression of iNOS-mediated Akt and ERK pathways: in-vitro and in-vivo studies. *Journal of Pharmacy and Pharmacology*.

[23] Lee J-D, Huh J-E, Jeon G (2009). Flavonol-rich RVHxR from *Rhus verniciflua* Stokes and its major compound fisetin inhibits inflammation-related cytokines and angiogenic factor in rheumatoid arthritic fibroblast-like synovial cells and in vivo models. *International Immunopharmacology*.

[24] Park DK, Lee YG, Park H-J (2013). Extract of *Rhus verniciflua* bark suppresses 2,4-dinitrofluorobenzene- induced allergic contact dermatitis. *Evidence-Based Complementary and Alternative Medicine*.

[25] Lee J-C, Lim K-T, Jang Y-S (2002). Identification of *Rhus verniciflua* Stokes compounds that exhibit free radical scavenging and anti-apoptotic properties. *Biochimica et Biophysica Acta—General Subjects*.

[26] Lim K-T, Hu C, Kitts DD (2001). Antioxidant activity of a *Rhus verniciflua* Stokes ethanol extract. *Food and Chemical Toxicology*.

[27] Ko J-H, Lee S-J, Lim K-T (2005). 36 kDa Glycoprotein isolated from *Rhus verniciflua* Stokes fruit has a protective activity to glucose/glucose oxidase-induced apoptosis in NIH/3T3 cells. *Toxicology in Vitro*.

[28] Jung CH, Jun C-Y, Lee S, Park C-H, Cho K, Ko S-G (2006). *Rhus verniciflua* stokes extract: radical scavenging activities and protective effects on H_2_O_2_-induced cytotoxicity in macrophage RAW 264.7 cell lines. *Biological and Pharmaceutical Bulletin*.

[29] Lee J-H, Lee H-J, Lee H-J (2009). *Rhus verniciflua* Stokes prevents cisplatin-induced cytotoxicity and reactive oxygen species production in MDCK-I renal cells and intact mice. *Phytomedicine*.

[30] Liu CS, Nam TG, Han MW (2013). Protective effect of detoxified *Rhus verniciflua* stokes on human keratinocytes and dermal fibroblasts against oxidative stress and identification of the bioactive phenolics. *Bioscience, Biotechnology, and Biochemistry*.

[31] Kang SY, Kang J-Y, Oh M-J (2012). Antiviral activities of flavonoids isolated from the bark of *Rhus verniciflua* stokes against fish pathogenic viruses in vitro. *Journal of Microbiology*.

[32] Cho N, Choi JH, Yang H (2012). Neuroprotective and anti-inflammatory effects of flavonoids isolated from *Rhus verniciflua* in neuronal HT22 and microglial BV2 cell lines. *Food and Chemical Toxicology*.

[33] Kim S, Park S-E, Sapkota K, Kim M-K, Kim S-J (2011). Leaf extract of *Rhus verniciflua* Stokes protects dopaminergic neuronal cells in a rotenone model of Parkinson’s disease. *Journal of Pharmacy and Pharmacology*.

[34] Byung CP, Yong SL, Park H-J (2007). Protective effects of fustin, a flavonoid from *Rhus verniciflua* stokes, on 6-hydroxydopamine-induced neuronal cell death. *Experimental and Molecular Medicine*.

[35] Sapkota K, Kim S, Kim M-K, Kim S-J (2010). A detoxified extract of *Rhus verniciflua* stokes upregulated the expression of BDNF and GDNF in the rat brain and the human dopaminergic cell line SH-SY5Y. *Bioscience, Biotechnology and Biochemistry*.

[36] Sapkota K, Kim S, Park S-E, Kim S-J (2011). Detoxified extract of *Rhus verniciflua* stokes inhibits rotenone-induced apoptosis in human dopaminergic cells, SH-SY5Y. *Cellular and Molecular Neurobiology*.

[37] Jeon WK, Lee JH, Kim HK (2006). Anti-platelet effects of bioactive compounds isolated from the bark of *Rhus verniciflua* Stokes. *Journal of Ethnopharmacology*.

[38] Song D-G, Lee JY, Lee EH (2010). Inhibitory effects of polyphenols isolated from *Rhus verniciflua* on Aldo-keto reductase family 1 B10. *BMB Reports*.

[39] Lee J, Lim K-T (2010). Plant-originated glycoprotein (36kDa) suppresses interleukin-4 and-10 in bisphenol Astimulated primary cultured mouse lymphocytes. *Drug and Chemical Toxicology*.

[40] Gupta SC, Kim JH, Prasad S, Aggarwal BB (2010). Regulation of survival, proliferation, invasion, angiogenesis, and metastasis of tumor cells through modulation of inflammatory pathways by nutraceuticals. *Cancer and Metastasis Reviews*.

[41] Hanahan D, Weinberg RA (2011). Hallmarks of cancer: the next generation. *Cell*.

[42] Gupta SC, Kim JH, Prasad S, Aggarwal BB (2012). Chronic inflammation and cancer: a matter of lifestyle. *Chronic Inflammation: Molecular Pathophysiology, Nutritional and Therapeutic Interventions*.

[43] Hansen I, Klimek L, Mösges R, Hörmann K (2004). Mediators of inflammation in the early and the late phase of allergic rhinitis. *Current Opinion in Allergy and Clinical Immunology*.

[44] Jung CH, Zhou S, Ding GX (2006). Antihyperglycemic activity of herb extracts on streptozotocin-induced diabetic rats. *Bioscience, Biotechnology and Biochemistry*.

[45] Oh P-S, Lee S-J, Lim K-T (2006). Hypolipidemic and antioxidative effects of the plant glycoprotein (36 kDa) from *Rhus verniciflua* stokes fruit in triton WR-1339-induced hyperlipidemic mice. *Bioscience, Biotechnology and Biochemistry*.

[46] Choi K-C, Chung W-T, Kwon J-K (2011). Chemoprevention of a flavonoid fraction from *Rhus verniciflua* Stokes on aflatoxin B1-induced hepatic damage in mice. *Journal of Applied Toxicology*.

[47] Ko J-H, Lee S-J, Lim K-T (2006). *Rhus verniciflua* Stokes glycoprotein (36 kDa) has protective activity on carbon tetrachloride-induced liver injury in mice. *Environmental Toxicology and Pharmacology*.

[48] Lim K-T, Lee S-J, Heo K-S, Lim K (2003). Effects of glycoprotein isolated from *Rhus verniciflua* stokes on TPA-induced apoptosis and production of cytokines in cultured mouse primary splenocytes. *Toxicology Letters*.

[49] Lee SH, Nan J-X, Zhao YZ (2003). The chalcone butein from *Rhus verniciflua* shows antifibrogenic activity. *Planta Medica*.

[50] Lee S-H, Choi W-C, Yoon S-W (2009). Impact of standardized *Rhus verniciflua* stokes extract as complementary therapy on metastatic colorectal cancer: a Korean single-center experience. *Integrative Cancer Therapies*.

[51] Lee S-H, Choi W-C, Kim K-S, Park J-W, Lee S-H, Yoon S-W (2010). Shrinkage of gastric cancer in an elderly patient who received *Rhus verniciflua* stokes extract. *Journal of Alternative and Complementary Medicine*.

[52] Kim HR, Kim KS, Jung HS, Choi WC, Eo WK, Cheon SH (2010). A case of recurred hepatocellular carcinoma refractory to doxorubicin after liver transplantation showing response to herbal medicine product, *Rhus verniciflua* stokes extract. *Integrative Cancer Therapies*.

[53] Lee SK, Jung HS, Eo WK, Lee SY, Kim SH, Shim BS (2010). *Rhus verniciflua* Stokes extract as a potential option for treatment of metastatic renal cell carcinoma: report of two cases. *Annals of Oncology*.

[54] Lee S, Kim K, Jung H (2012). Efficacy and safety of standardized allergen-removed *Rhus verniciflua* Stokes extract in patients with advanced or metastatic pancreatic cancer: a Korean single-center experience. *Oncology*.

[55] Lee S-H, Kim K-S, Choi W-C, Yoon S-W (2009). Successful outcome of advanced pulmonary adenocarcinoma with malignant pleural effusion by the standardized *Rhus verniciflua* stokes extract: a case study. *Explore: The Journal of Science and Healing*.

[56] Lee J, Chae J, Lee S (2013). The efficacy and safety of standardized allergen-removed *Rhus verniciflua* extract as maintenance therapy after first-line chemotherapy in patients with advanced non-small cell lung cancer. *The American Journal of Chinese Medicine*.

[57] Lee EH, Song D-G, Lee JY, Pan C-H, Um BH, Jung SH (2008). Inhibitory effect of the compounds isolated from *Rhus verniciflua* on aldose reductase and advanced glycation endproducts. *Biological and Pharmaceutical Bulletin*.

[58] Jeong G-S, Lee D-S, Song M-Y (2011). Butein from *Rhus verniciflua* protects pancreatic *β* cells against cytokine-induced toxicity mediated by inhibition of nitric oxide formation. *Biological and Pharmaceutical Bulletin*.

[59] Khan N, Adhami VM, Afaq F, Mukhtar H (2012). Butein induces apoptosis and inhibits prostate tumor growth in vitro and in vivo. *Antioxidants and Redox Signaling*.

[60] Lu M, Wang S, Han X, Lv D (2013). Butein inhibits NF-*κ*B activation and reduces infiltration of inflammatory cells and apoptosis after spinal cord injury in rats. *Neuroscience Letters*.

[61] Gábor M, Eperjessy E (1966). Antibacterial effect of fisetin and fisetinidin. *Nature*.

[62] Schwartz AG, Rate WR (1979). Inhibition of aflatoxin B1-induced cytotoxicity and binding to DNA in cultured rat liver cells by naturally occurring flavones. *Journal of Environmental Pathology and Toxicology*.

[63] Middleton E, Drzewiecki G (1984). Flavonoid inhibition of human basophil histamine release stimulated by various agents. *Biochemical Pharmacology*.

[64] Nakadate T, Yamamoto S, Aizu E, Kato R (1984). Effects of flavonoids and antioxidants on 12-O-tetradecanoyl-phorbol-13-acetate-caused epidermal ornithine decarboxylase induction and tumor promotion in relation to lipoxygenase inhibition by these compounds. *Gann, The Japanese Journal of Cancer Research*.

[65] Ferriola PC, Cody V, Middleton E (1989). Protein kinase C inhibition by plant flavonoids. Kinetic mechanisms and structure-activity relationships. *Biochemical Pharmacology*.

[66] Francis AR, Shetty TK, Bhattacharya RK (1989). Modulating effect of plant flavonoids on the mutagenicity of N-methyl-N′-nitro-N-nitrosoguanidine. *Carcinogenesis*.

[67] Yamashita Y, Kawada S-Z, Nakano H (1990). Induction of mammalian topoisomerase II dependent DNA cleavage by nonintercalative flavonoids, genistein and orobol. *Biochemical Pharmacology*.

[68] Tzeng S-H, Ko W-C, Ko F-N, Teng C-M (1991). Inhibition of platelet aggregation by some flavonoids. *Thrombosis Research*.

[69] Soliman KFA, Mazzio EA (1998). In vitro attenuation of nitric oxide production in C6 astrocyte cell culture by various dietary compounds. *Proceedings of the Society for Experimental Biology and Medicine*.

[70] Park JB (1999). Flavonoids are potential inhibitors of glucose uptake in U937 cells. *Biochemical and Biophysical Research Communications*.

[71] Joussen AM, Rohrschneider K, Reichling J, Kirchhof B, Kruse FE (2000). Treatment of corneal neovascularization with dietary isoflavonoids and flavonoids. *Experimental Eye Research*.

[72] Zhang M, Zhang J-P, Ji H-T, Wang J-S, Qian D-H (2000). Effect of six flavonoids on proliferation of hepatic stellate cells in vitro. *Acta Pharmacologica Sinica*.

[73] Chen Y-C, Shen S-C, Lee W-R (2002). Wogonin and fisetin induction of apoptosis through activation of caspase 3 cascade and alternative expression of p21 protein in hepatocellular carcinoma cells SK-HEP-1. *Archives of Toxicology*.

[74] Hirano T, Higa S, Arimitsu J (2004). Flavonoids such as luteolin, fisetin and apigenin are inhibitors of interleukin-4 and interleukin-13 production by activated human basophils. *International Archives of Allergy and Immunology*.

[75] Jeon TI, Park JW, Ahn J, Jung CH, Ha TY (2013). Fisetin protects against hepatosteatosis in mice by inhibiting miR-378. *Molecular Nutrition & Food Research*.

[76] Léotoing L, Wauquier F, Guicheux J, Miot-Noirault E, Wittrant Y, Coxam V (2013). The polyphenol fisetin protects bone by repressing NF-*κ*B and MKP-1-dependent signaling pathways in osteoclasts. *PLoS ONE*.

[77] Prakash D, Gopinath K, Sudhandiran G (2013). Fisetin enhances behavioral performances and attenuates reactive gliosis and inflammation during aluminum chloride-induced neurotoxicity. *NeuroMolecular Medicine*.

[78] Prasath GS, Sundaram CS, Subramanian SP (2013). Fisetin averts oxidative stress in pancreatic tissues of streptozotocin-induced diabetic rats. *Endocrine*.

[79] Baker ME, Medlock KL, Sheehan DM (1998). Flavonoids inhibit estrogen binding to rat alpha-fetoprotein. *Proceedings of the Society for Experimental Biology and Medicine*.

[80] Sugihara N, Arakawa T, Ohnishi M, Furuno K (1999). Anti- and pro-oxidative effects of flavonoids on metal-induced lipid hydroperoxide-dependent lipid peroxidation in cultured hepatocytes loaded with *α*-linolenic acid. *Free Radical Biology and Medicine*.

[81] Takano-Ishikawa Y, Goto M, Yamaki K (2003). Inhibitory effects of several flavonoids on E-selectin expression on human umbilical vein endothelial cells stimulated by tumor necrosis factor-*α*. *Phytotherapy Research*.

[82] Shimada H, Miura K, Imamura Y (2006). Characteristics and inhibition by flavonoids of 20*α*-hydroxysteroid dehydrogenase activity in mouse tissues. *Life Sciences*.

[83] Park H-H, Lee S, Son H-Y (2008). Flavonoids inhibit histamine release and expression of proinflammatory cytokines in mast cells. *Archives of Pharmacal Research*.

[84] Devi MA, Das NP (1993). In vitro effects of natural plant polyphenols on the proliferation of normal and abnormal human lymphocytes and their secretions of interleukin-2. *Cancer Letters*.

[85] Jin C-H, Shin E-J, Park J-B (2009). Fustin flavonoid attenuates *β*-amyloid (1-42)-induced learning impairment. *Journal of Neuroscience Research*.

[86] Westenburg HE, Lee K-J, Lee SK (2000). Activity-guided isolation of antioxidative constituents of Cotinus coggygria. *Journal of Natural Products*.

[87] Song M-Y, Jeong G-S, Kwon K-B (2010). Sulfuretin protects against cytokine-induced *β*-cell damage and prevents streptozotocin-induced diabetes. *Experimental and Molecular Medicine*.

[88] Song M-Y, Jeong G-S, Lee H-S (2010). Sulfuretin attenuates allergic airway inflammation in mice. *Biochemical and Biophysical Research Communications*.

[89] Lee K-W, Chung K-S, Seo J-H (2012). Sulfuretin from heartwood of *Rhus verniciflua* triggers apoptosis through activation of Fas, Caspase-8, and the mitochondrial death pathway in HL-60 human leukemia cells. *Journal of Cellular Biochemistry*.

[90] Lee Y-R, Hwang J-K, Koh H-W (2012). Sulfuretin, a major flavonoid isolated from *Rhus verniciflua*, ameliorates experimental arthritis in mice. *Life Sciences*.

[91] Kim J-M, Noh E-M, Kwon K-B (2013). Suppression of TPA-induced tumor cell invasion by sulfuretin via inhibition of NF-*κ*B-dependent MMP-9 expression. *Oncology Reports*.

[92] Poudel S, Song J, Jin E-J, Song K (2013). Sulfuretin-induced miR-30C selectively downregulates cyclin D1 and D2 and triggers cell death in human cancer cell lines. *Biochemical and Biophysical Research Communications*.

[93] Richter M, Ebermann R, Marian B (1999). Quercetin-induced apoptosis in colorectal tumor cells: possible role of EGF receptor signaling. *Nutrition and Cancer*.

[94] Fiander H, Schneider H (2000). Dietary ortho phenols that induce glutathione S-transferase and increase the resistance of cells to hydrogen peroxide are potential cancer chemopreventives that act by two mechanisms: the alleviation of oxidative stress and the detoxification of mutagenic xenobiotics. *Cancer Letters*.

[95] Mastuda H, Morikawa T, Ueda K, Managi H, Yoshikawa M (2002). Structural requirements of flavonoids for inhibition of antigen-induced degranulation, TNF-*α* and IL-4 production from RBL-2H3 cells. *Bioorganic and Medicinal Chemistry*.

[96] Hendriks JJA, De Vries HE, Van Der Pol SMA, Van Den Berg TK, Van Tol EAF, Dijkstra CD (2003). Flavonoids inhibit myelin phagocytosis by macrophages; a structure-activity relationship study. *Biochemical Pharmacology*.

[97] Monasterio A, Urdaci MC, Pinchuk IV, López-Moratalla N, Martínez-Irujo JJ (2004). Flavonoids induce apoptosis in human leukemia U937 cells through caspase- and caspase-calpain-dependent pathways. *Nutrition and Cancer*.

[98] Kim H, Park B-S, Lee K-G (2005). Effects of naturally occurring compounds on fibril formation and oxidative stress of *β*-amyloid. *Journal of Agricultural and Food Chemistry*.

[99] Won JL, Shim J-Y, Zhu BT (2005). Mechanisms for the inhibition of DNA methyltransferases by tea catechins and bioflavonoids. *Molecular Pharmacology*.

[100] Lyu S-Y, Rhim J-Y, Park W-B (2005). Antiherpetic activities of flavonoids against herpes simplex virus type 1 (HSV-1) and type 2 (HSV-2) in vitro. *Archives of Pharmacal Research*.

[101] Ichimatsu D, Nomura M, Nakamura S (2007). Structure-activity relationship of flavonoids for inhibition of epidermal growth factor-induced transformation of JB6 CI 41 cells. *Molecular Carcinogenesis*.

[102] Lin C-W, Hou W-C, Shen S-C (2008). Quercetin inhibition of tumor invasion via suppressing PKC*δ*/ERK/ AP-1-dependent matrix metalloproteinase-9 activation in breast carcinoma cells. *Carcinogenesis*.

[103] Resende FA, de Oliveira AP, de Camargo MS, Vilegas W, Varanda EA (2013). Evaluation of estrogenic potential of flavonoids using a recombinant yeast strain and MCF7/BUS cell proliferation assay. *PLoS ONE*.

[104] Scartozzi M, Pistelli M, Bittoni A (2010). Novel perspectives for the treatment of gastric cancer: from a global approach to a personalized strategy. *Current Oncology Reports*.

[105] Azim J, de Azambuja E, Colozza M, Bines J, Piccart MJ (2011). Long-term toxic effects of adjuvant chemotherapy in breast cancer. *Annals of Oncology*.

[106] Cutuli B, Kanoun S, Tunon De Lara C (2012). Breast cancer occurred after Hodgkin’s disease: clinico-pathological features, treatments and outcome: analysis of 214 cases. *Critical Reviews in Oncology/Hematology*.

[107] Burris HA, Moore MJ, Andersen J (1997). Improvements in survival and clinical benefit with gemcitabine as first- line therapy for patients with advanced pancreas cancer: a randomized trial. *Journal of Clinical Oncology*.

[108] Moore MJ, Goldstein D, Hamm J (2007). Erlotinib plus gemcitabine compared with gemcitabine alone in patients with advanced pancreatic cancer: a phase III trial of the National Cancer Institute of Canada Clinical Trials Group. *Journal of Clinical Oncology*.

[109] Cunningham D, Chau I, Stocken DD (2009). Phase III randomized comparison of gemcitabine versus gemcitabine plus capecitabine in patients with advanced pancreatic cancer. *Journal of Clinical Oncology*.

[110] Buckkingham J (1994). *Dictionary of Natural Products*.

[111] Yit CC, Das NP (1994). Cytotoxic effect of butein on human colon adenocarcinoma cell proliferation. *Cancer Letters*.

[112] Tilton B (2004). *Wilderness First Responder: How to Recognize, Treat, and Prevent Emergencies in the Backcountry*.

[113] Ehling D (2001). Oriental medicine: an introduction. *Alternative Therapies in Health and Medicine*.

